# Development of Sport Courage Scale

**DOI:** 10.2478/v10078-012-0055-z

**Published:** 2012-07-04

**Authors:** Erkut Konter, Johan Ng

**Affiliations:** 1 Buca Educational Faculty, Dokuz Eylül University, İzmir, TURKEY.; 2 University of Birmingham, School of Sport and Exercise Sciences, UK.

**Keywords:** courage, sport, scale development, risk

## Abstract

While theory and practice of sport have much to say about fear, stress and anxiety, they have little to say about courage. Therefore, the purpose of this study was to develop a Sport Courage Scale. Data were collected from two groups of male and female athletes aged from 13 to 22 in different individual and team sports. The first set of data (N = 380) was analyzed by exploratory factor analysis, and the second set of data (N = 388) was analyzed by confirmatory factor analysis. Analyses revealed a 5-factor structure of Sport Courage Scale that supported factorial validity and reliability of scale scores. These factors were labelled: “Determination”, “Mastery”, “Assertiveness”, “Venturesome”, and “Self-Sacrifice Behaviour”. Finally, evidence of test-retest reliability of scale scores was supported based on responses from 75 athletes. However, more research is needed to further improve the Sport Courage Scale.

## Introduction

Noted sport psychologist Gould (2002) reported that sport psychology can help the field of general psychology move into a millennium of positive psychology to facilitate functioning and performance enhancement in sport. However, theory and practice of sport in general, and sport psychology in particular, have much to say about stress, anxiety and fear and they have little to say about courage in sport from the perspective of positive psychology (Corlett, 2002).

For the ancient Greeks, courage was a part of virtuous living. Virtues are the core characteristics valued by moral philosophers: wisdom, courage, humanity, justice, temperance, and transcendence (Park and Peterson, 2004). Plato wrote a dialogue, Laches, on virtuous living. Courage was a part of Aritotle’s broader notion of “excellence of character” in his works on ethics. Courage came easily to these ancients because their very notion of living included courage. In contrast, courage today is not perceived as a virtue and a purpose in itself to achieve excellent character, but a skill and an instrument to benefit from (Corlett, 2002).

When asked to describe a courageous action, people take it personally and overwhelmingly describe courageous action with a successful outcome (Pury et al., 2007). In addition, Pury and Hensel (2010) found that successful actions were rated as more courageous than unsuccessful outcomes, although this effect was attenuated for external attributions. Moreover, Kilmann et al. (2010) emphasized that acts of courage might have a dramatic impact on employees and the long-term success of an organization. All of these findings related to courage and success could be important for studying sport courage.

Various authors have suggested a number of different definitions and concepts of courage (Lopez, 2007). Park and Peterson (2004) identified courage as an important part of character strength among youth. For Park and Peterson, courage is an emotional strength that involves the exercise of will to accomplish goals in the face of opposition, external or internal, which consists of bravery, industry/perseverance (persistence), authenticity/honesty (integrity) and zest (vitality). In addition, Mavroudis (2003) put forward that courage is an individual’s selfless pursuit of a moral good while risking personal harm, injury, or death. Moreover, Mavroudis (2003) also emphasized that a) courage can be inversely related to knowledge, b) courage is needed to transform an action of uncertainty into an action of confidence through knowledge, c) less courage is needed, when someone possesses more knowledge, even the task is difficult to be performed, d) courage is necessary when there is little or no knowledge to perform a procedure or to try an unproven solution, e) courage should be dependent on when it is necessary, and f) courage could be relative as competence and knowledge are not static.

Woodard (2004) indicated that courage is an integral part of the existential concept of authenticity. Woodard and Pury (2007) defined courage as “the voluntary willingness to act, with or without varying levels of fear, in response to a threat to achieve an important, perhaps moral, outcome or goal”. These definitions acknowledge that fear may or may not be present to any significant degree for an act to be considered courageous, and makes evident the two generally agreed upon components of courage, threat and worthy or important outcome.

Kilmann et al. (2010) reviewed courage concepts and definitions and suggested that courageous actions in organizations include five essential properties: 1) free choice for deciding whether to act (versus being coerced), 2) significant risk of being harmed, 3) assessment that risk is a reasonable and contemplated act which is considered justifiable (not foolhardy), 4) pursuit of worth aims, and 5) proceeding with mindful actions despite fear. Martin (2011) studied academic courage and defined it as “perseverance in the face of academic difficulty and fear”. In addition, sport courage was specifically defined as “natural and developed, interactional and perceptual concept between person and situation, and the task at hand that enables person to move in competence, mastery, determination, assertiveness, venturesome and sacrificial (altruistic) behaviour on voluntary basis and in danger circumstances” (Konter et al., 2013). As with most judgements of human nature, grey areas exist that warrant definitions, assessment of circumstances, and weighting options (Mavroudis, 2003), as it might be the case between the suggested definitions of courage.

Martin (2011) studied courage in the classroom predicting academic performance (planning, task management, self-handicapping, disengagement, class participation, employment of school, and positive intentions) and found a four-factor solution comprising courage, confidence, avoidance, and helpless orientation. Results of the research also indicated that courage and confidence are not significantly different on some academic measures (including performance) but that across the bulk of measures confidence is more adaptive. However, courage was unambiguously more adaptive than avoidance and helpless orientations across all outcome measures. Although confidence yields the most positive educational outcomes, courage can be considered an educationally effective response in the face or presence of fear. In addition, Woodard (2004) found that courage did not add to the amount of the variance of physiological health accounted for by hardiness. Hardiness was suggested to provide the courage and motivation to do the hard, strategic work, and turning stressful circumstances from potential disasters into growth opportunities (Salvtore, 2006). Moreover, Schmidt and Koselka (2000) developed The Courage Scale, which includes 7 items with the first three items assessing “general courage”, and the last four items assessing “panic specific courage”.

Woodard (2004) proposed a three sequential interrelated conceptual model of courage. According to Woodard; a) fear is a prerequisite for courage, b) fear is the result of a perception of vulnerability, c) fear is established by the accurate or inaccurate evaluation of a threat as outweighing the personal resources of the individual, d) perception of the meaningfulness by the individual is important to react or not to react to the threat, e) managing the physiological reactions to fear is vital, f) the benefits of engaging the threat without sufficient resources would need to outweigh the potential costs, g) courageous behaviour happens as a result of high perception of meaningfulness to react. In addition, Hannah et al. (2007) suggested a model outlining the subjective experience of courage. They proposed various positive psychological states and traits to reduce the level of fear experienced when facing risk, and create a courageous mindset. Moreover, Konter et al. (2013) put forward a sport specific courage model emphasizing the interactions between factors including situations (e.g., risk, danger, fear at present), personal differences (e.g., personality traits, experience and knowledge of the athlete), sport (e.g., individual and team sports, contact and non-contacted sports), and the task at hand (e.g., take a decisive penalty kick at the last second of a soccer game or free throw in basketball). Therefore, the concept of sport courage is suggested to be a dynamic and transformational process changing (positive or negative, increase or decrease) as a result of aforementioned interactions over time (Konter et al., 2013; Mavroudis, 2003).

The latest courage research covered such subjects as courage in the classroom (Martin, 2011), courageous actions and success (Pury and Hensel, 2010), organizational courage (Kilmann et al., 2010), fear and courage in children (Moris, 2009), civil courage, implicit theories, and measurement (Greitemeyer et al., 2007; Rate et al., 2007), the subjective act and experience of courage (Hannah et al., 2007), courageous actions, and general as well as personal courage (Pury and Kowaski, 2007; Pury et al., 2007), courageous altruism (Fagin-Jones and Midlarsky, 2007), the construct of courage and measurement (Woodard and Pury, 2007), hardiness and the concept of courage (Salvtore, 2006; Woodard, 2004), and partnership in courage (Mavroudis, 2003). Although researchers have concentrated on concepts and measurements related to courage in various aforementioned fields, there is a paucity of research to understand sport courage.

Athletes have been known to display many forms of courage by virtue of their basic human behaviour, intellectual, cognitive, physical, emotional and social fortitude, and resolve while taking care of their opponents (sportsmanship). In addition, athletes who performed their best breaking records, took relatively high risks and challenged with their strong opponents in stressful situations, are all exemplars of courage (Konter et al., 2010).

There is obviously a lack of research related to measurement of courage in sport in order to ask various interesting research questions. Therefore, this study involved an exploration of courage in sport and the development of psychometrically sound measurement of courage in sport.

## Material and Methods

### Participants

In order to examine the factor structure of the newly developed scale, data were collected from two separate groups of athletes. All participants of the study were recruited through sports clubs and schools. The first sample consisted of 380 Turkish athletes (age = 15.68 ± 2.23 yrs) from a diversity of team and individual sports (see Table 1 for details). The second sample included a separate group of 388 athletes (age = 15.69 ± 2.30 yrs). Another 75 athletes were then recruited, their responses were used to examine the test-retest reliability of scale scores.

### Instrument Development: The Sport Courage Scale (SCS):

Prior to the initial item creation, meetings were held with 50 coaches, teachers, and university lecturers specialised in sports to note down their views, interpretations and examples of courage in sport. Group discussions were held with 100 student athletes to gain insights of the topic from an athlete’s point of view. Brief questionnaires containing three open-ended questions were administered to 400 athletes to gather more examples of how sport courage was seen and interpreted. Based on the results of these, a pool of 300 items describing feelings and experiences regarding sport courage was created in Turkish. The items were then revised by six specialists in sport psychology. Similar items or those that were not considered measuring sport courage were eliminated. The final item pool consisted of 79 items.

A pilot test was conducted to review wordings and understandability of the items. The 79 items were administered to 100 athletes (50 male, 50 female) after receiving informed consent from them. Responses were made using a 5-point Likert scale. All items were deemed understandable and feasible to athletes, and they were subsequently used to form the initial version of the SCS.

### Procedures and Data Analysis

The initial SCS was administered to the first sample of athletes, together with questionnaires on their demographics (e.g., gender, age, main sport). Permission for data collection was granted by coaches, teachers, and administrators for sport clubs. All athletes also provided informed consent to take part in the study. Responses obtained from this sample were examined by exploratory factor analysis (EFA) using SPSS version 15.0. The number of factors to be extracted was determined using the eigenvalue > 1 rule, and a varimax rotation was applied. Variables were considered within one factor if the corresponding loadings exceeded .40. Items that did not belong to any factors, or were loaded on more than a single factor were eliminated from the scale.

The retained items were then administered to the second group of participants together with the same demographic questions. Based on the results obtained in the EFA, confirmatory factor analyses (CFA) were conducted on scale scores using Mplus version 5.21 (Muthén and Muthén, 2008). In order to provide a comprehensive indication of model fit, several different fit indices were examined. First, the overall test of model fit was determined by the chi-square test. Since the chi-square statistic is sensitive to sample size, possibly leading to the rejection of adequate models, more weight was placed on the consideration of other fit indices. These indices include Comparative Fit Index (CFI), Tucker Lewis Index (TLI), Root Mean Square Error of Approximation (RMSEA), and the Standardized Root Mean Square Residual (SRMR). Traditionally, a good model fit is supported when CFI and TLI values exceeded .90, while that of RMSEA was smaller than .08 (Bentler and Bonett, 1980). Hu and Bentler (1999) suggested using cutoff values of .95 for CFI and TLI instead, while RMSEA and SRMR should not exceed .06 and .08 respectively. These cutoff values were used as the criteria of very good model fit in this study. Item scores with factor loadings < .4 were eliminated from the analyses, and modification indices were used to identify cross-loadings. Finally, to test whether the measured factors belonged to a single broader construct of courage, a second order CFA was conducted on the data. Model fit was evaluated using the same criteria mentioned above. Cronbach alphas of scale score in the final model were also obtained to examine their internal consistencies.

Finally, the test-retest reliability of scale scores was examined. Specifically, 75 athletes completed the two administrations of SCS over three weeks’ time. Intraclass correlations (ICC) between the corresponding subscale scores and the total scores were computed.

## Results

The EFA extracted five factors using the eigenvalue > 1 rule. The scores of 40 items did not load on any factor, while another 4 items loaded on more than a single factor. These items were deemed problematic and removed from the scale. The modified scale contained 5 factors and 35 items explaining 47 % of total variance (please see Table 2). KMO and Barlett’s Test revealed the following results with 5 factor and 35 items: Kaiser-Meyer-Olkin Measure of Sampling Adequacy; .89, Barlett’s Test of Sphericity Chi-Square; 4233.07, df; 595, Sig; .000. In addition, based on the contents of the remaining items, the factors were named as: “Determination” (DT, 10 items, M=39.52 and SD=4.40), “Mastery” (as a more specific and an important resource of Self-confidence, Vealey, et al., 1998) (MT, 8 items, M=23.19 and SD=6.40), “Assertiveness” (AT; 8 items, M=28.17 and SD=3.88), “Venturesome” (VS, 5 items, M=16.47 and SD=3.07), and “Sacrifice Behaviours” (SB; 4 items, M=16.47 and SD=2.74). See detailed results of the EFA in Table 2. Moreover, the EFA indicated that factor loadings of the 5 factors change between .41 and .78 (.60 and .75 for MT; .43 and .65 for DT; .41 and .70 for AT; .41 and .78 for VS; .45 and .73 for SB).

Based on the EFA results, a CFA was conducted on the responses from the second sample using maximum likelihood, and by allowing the items to load on their corresponding factors only. The initial model had a good fit to the data: χ^2^ (550) = 733.62, *p* < .01, CFI = .93, TLI = .93, RMSEA = .03, SRMR = .05, but one item (item 66) had a loading < .4, modification indices suggested that 4 items cross-loaded on a multiple factors. These items were eliminated one by one, and the resultant model contained 31 items. This model had a very good fit to the data: χ^2^ (424) = 535.50, *p* < .01, CFI = .95, TLI = .95, RMSEA = .03, SRMR = .05.

In the modified model, the factor correlations between DT and AT were found to be very strong (r = .82). Based on suggestions by Thompson and Daniel (1996), an alternative nested model was tested by fixing the covariance between the two factors to 1.0 to check whether those two factors should be combined to form a single factor. The change in model fit was examined using changes in CFIs (ΔCFI; Cheung and Rensvold, 2002), where a decrease of more than .01 in the CFI statistics (i.e., ΔCFI < −.01) would suggest a reduced model fit. The results showed that model fit was reduced when the covariances of the two factors were set to 1 (ΔCFI = −.15), hence the two factors were not combined into a single factor.

**Figure 1:**
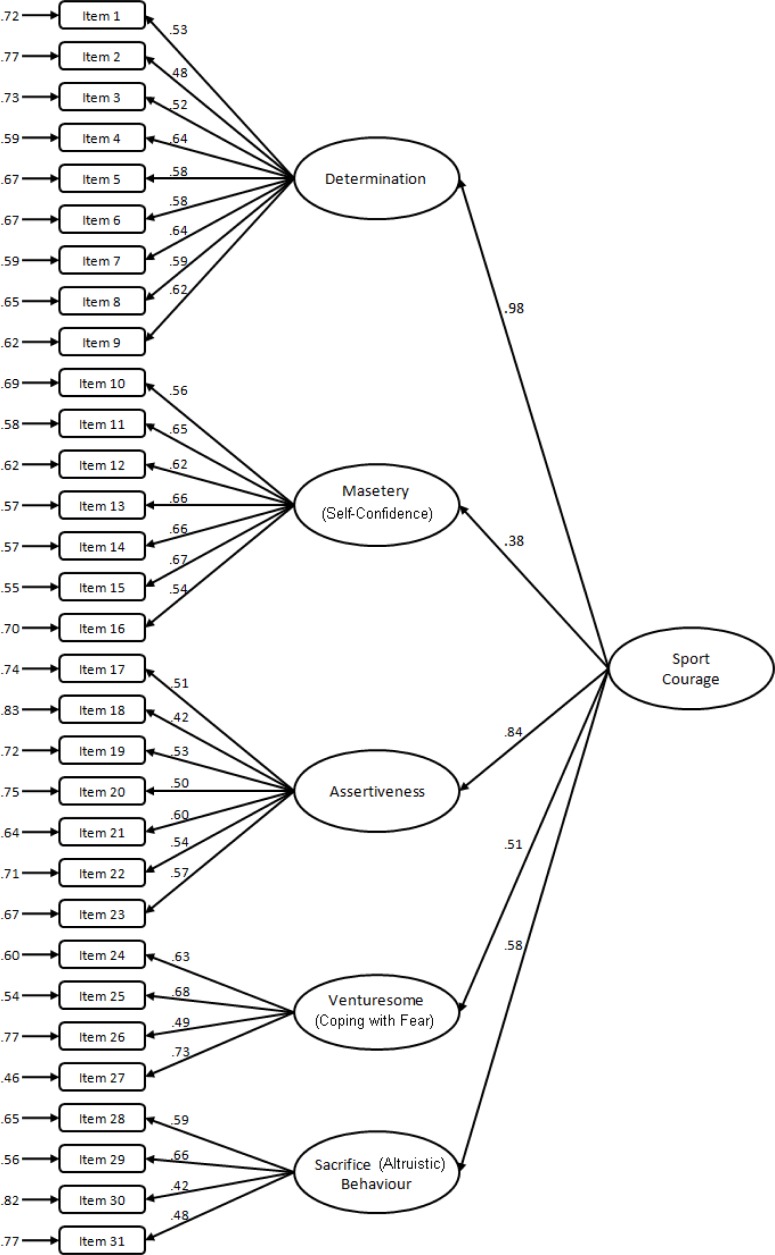
Five Factor Model of SCS Scores

A CFA was then conducted by loading the factors onto a second-order factor of sport courage. The results still suggested a good fit: χ^2^ (429) = 584.32, *p* < .01, CFI = .93, TLI = .93, RMSEA = .03, SRMR = .06 (Figure 1). The Cronbach alphas for scales scores were: DT = .82, MT = .82, AT = .72, VS = .72, SB = .61.

Finally, using ICC calculated based on responses from 75 athletes, test-retest reliability of SCS scores were generally supported: DT = .73; MT = .77; AT = .67; VS = .74; SB = .62; total SCS = .82.

## Discussion

The results supported a five-factor structure of SCS scores. CFA analyses indicated that the first factor of SCS is DT. This result supports Park and Peterson’ (2004) research related to factors of courage and persistency (perseverance and industry).

Determination is from Latin meaning limiting and hence the establishment of limits and boundaries, which is defined as “a trait of personality characterized by a tendency to push onward one’s goal despite barriers and hardships, it also means “reaching of a conclusion, the making of a decision” (Dictionary of Psychology, Reber, 1995).

Therefore, DT related items of SCS incorporates items such as “I perform to the best of my ability no matter how negative the current conditions are in my sport”, “Even when under pressure I do not lose sight of my goals in my sport”.

The second factor of SCS is MT (as an important source of self-Confidence, Vealey et al., 1998). Vealey et al. (1998) found various sources of self-confidence including MT. Sport psychologists define self-confidence as “the belief that athlete can successfully perform a desired behaviour” (Weinberg and Gould, 2007; Vealey, 1986), and “on occasion, it could be bold” (Cashmore, 2008). Vealey et al. (1998) suggested that MT, as a source of self-confidence, involves performing well, improving and achieving personal goals”. The accomplishment or application of a skill is known mastery, a term taken from the old French word “maistre”, in the form of the Latin magister, a commanding superior. This term is especially used in sports related skill execution” (Cashmore, 2008). In addition, Reber (1995) in the dictionary of psychology defined mastery as simply “achieving some pre-set (and usually high) level of functioning in some task”. Vealey and Chase (2008) revealed that there are specifically a few types of self-confidence (including mastery) within sport. For example, physical, psychological, perceptual, physical fitness and training status, ability to improve one’s skill. In addition, Vealey and Chase (2008) also talked about resilient self-confidence in sport and they reported that “elite athletes identified not just confidence but a rather resilient confidence in the form of unshakable self-belief”. This might be related to DT as a factor of sport courage in the present study. Therefore, MT related items of SCS includes reversed items such as “My doubts regarding my abilities prevent me from succeeding in my sport”, “I become pessimistic when faced with difficult situations in my sport”.

The third factor of SCS is AT. Assertiveness is that “use of legitimate, acceptable physical force and the expenditure of an unusually high degree of effort to achieve an external goal, with no intent to injure” (Kent, 2005) and “sometimes showing a self-confident approach” (Cashmore, 2008). This might be a kind of vitality (zest) which was suggested by Park and Petersen (2004) as approaching life with energy and excitement. Therefore, exemplars of assertiveness’ items related to sport courage measured by SCS incorporate “I like to take the initiative in the face of difficulties in my sport”, “I assert myself even when facing hazardous situations in my sport”.

The fourth factor of SCS is VS. Above definitions of courage emphasized that one distinction of courage is relatively high risk taking behaviour which must be present in sport situations. Risk is from the Italian “risco” for “danger”, risk means exposure to jeopardy. It is a word that crops up a lot. In all sports, athletes often run risks; in some, they put their lives at risk (e.g., extreme sports). Exercise itself is a form of health risk management. So, sport and exercise are full of risk factors (Cashmore, 2008). While there may be economic risks associated with sport (e.g., gambling) and social risks (risk of one’s reputation and social status) of central concern has been the risk of physical injury (and death). A “culture of risks” in sport has been indentified largely in the context of the wide spread acceptance of playing through pain and injury (Malcolm, 2008). Therefore, it could be argued that courage involves relatively high risk situations (perceived by the athlete) rather than an ordinary sport life.

It might be suggested that courage is not fearlessness. Rather, it is coping with fear in the face of high risks or dangers. Therefore, VS involves coping with fear. Fear may be no more than the brief thoughts of physical injury that flash through the minds of rugby (or soccer) full back’s fleeting image of another broken nose as he prepares to dive on the ball at the feet of opposing players. In some sports the merest hind of fear might be enough to end careers. All players have doubts and fears, although some may be good at hiding them. Everyone is human and susceptible to fear, fatigue, and indecision (Karageorghis and Terry, 2011). The result of present research supports the studies related to coping with fear and courageous behaviour (Corlett, 2002; Kilmann et al., 2010; Konter et al., 2013; Martin, 2011; Woodard and Pury, 2007). Fear is “an emotion associated with an actual impending danger or evil”. It is often characterized by the subjective experience of discomfort and arousal. Fear can induce a kind of paralysis in some competitors so that they freeze in the face of a forbidding rival. It can also act as a friend causing exhilaration that facilitates optimum performance” (Cashmore, 2008). On one hand, fear can cause freeze, avoidance, insensibility, persistent anxiety, psychopathology, etc. On the other hand, it might affect exhilaration, aggression, difficulty in psyching up for opponents. In the face of potential threat, the experience of fear is appropriate, but there are many instances in which fear is disproportionately great or persists long after the removal of the threat (Giulia et al., 2002). Therefore, VS related items of SCS include items such as “I risk injury in order not to lose in my sport”, “Even when facing the possibility of injury, I perform to the best of my ability in my sport”.

The fifth and final factor of SCS is SB. Courage could involve SB related to irrational attitude, altruistic act in high risk situations. This supports the aforementioned Mavroudis’ (2003) definition of courage is that “an individual’s selfless pursuit of a moral good while risking personal harm, injury, or death. Therefore, sacrifice (altruistic) behaviour related items of SCS incorporates “I do not hesitate to compete, even when facing the possibility of defeat in my sport”, “I defend my beliefs until the end, even if this action could prove harmful to me in my sport”.

Despite the supportive validity evidence of SCS scores, researchers may want to treat results with care. In particular, the alpha coefficient for the SB subscale was moderate (α = .61). Researchers will have to re-examine this aspect of scale scores in future studies. The subscale currently contains only four items, and potentially more items could be added in order to improve the reliability of scale scores. Also, CFA results showed that MT had a rather low second-order loading (.38) onto the broad construct of sport courage. However, as discussed above, MT is an important aspect of sport courage and hence should be included within the scale from a theoretical point of view. Researchers using the scale in the future may want to re-examine this aspect of validity of SCS scores.

Establishing support for the validity and the reliability of SCS is an ongoing project. Initial results of the present study regarding measuring courage in sport seem to be encouraging with the outcomes obtained. Future researchers can concentrate on more specific models and measurements of courage in sport. For example, social courage, emotional courage, intellectual courage, physical courage, trait sport courage, state sport courage, general and specific sport courage etc.

Researchers could also pose interesting research questions, for example, whether significant differences exist between courage and various sports, gender, level of participation, playing positions, personality traits, and various measures of performance, health and satisfaction in sport or not. Moreover, future research is necessary to examine the validity of similar scales (criterion validity) with the factors of SCS for further validity support. For example, various scales of self-confidence, coping, achievement motivation, competitiveness, assertiveness, altruism, helping behavior, mental toughness, hardiness, risk taking or fear scales in sport.

## Conclusions

Results suggest that initial efforts to develop a sport courage scale are encouraging. The present research showed initial evidence supporting the validity and reliability of the SCS. Using a series of qualitative and quantitative methods, items of the SCS emerged with 5 factors, namely Mastery (Self-Confidence), Determination, Assertiveness, Venturesome (Coping With Fear, Risk Taking), and Self-Sacrifice Behavior (Altruism).

## Figures and Tables

**Table 1:** Descriptive Statistics of Participants

Variables	Sample 1		Sample 2	
	N	%	N	%
Total	380		388	
Gender				
Male	275	72.4	280	72.2
Female	104	27.4	107	27.6
Sports				
Football	171	45.0	176	45.4
Basketball	38	10.0	26	6.7
Handball	14	3.7	16	4.1
Volleyball	21	5.5	21	5.4
Wrestling	21	5.5	37	9.5
Boxing	9	2.4	7	1.8
Karate	12	3.2	12	3.1
Taekwondo	15	3.9	13	3.4
Track and field	18	4.7	24	6.2
Tennis	9	2.4	10	2.6
Badminton	7	1.8	4	1.0
Fencing	8	2.1	9	2.3
Sailing	7	1.8	10	2.6
Judo	7	1.8	6	1.5
Swimming	18	4.7	7	1.8
Diving	2	0.5	8	2.1
Triathlon	3	0.8	1	0.3
Level				
Professional	36	9.5	38	9.8
Amateur	344	90.5	350	90.2

**Table 2:** Results of Total Variance Explained By EFA

	Initial Eigenvalues			Extraction Sums of Squared Loadings			Rotation Sums of Squared Loadings		

Factor	Total	%Variance	%Cumulative	Total	%Variance	%Cumulative	Total	%Variance	%Cumulative
DT	7.90	22.59	22.59	7.91	22.59	22.59	4.12	11.77	11.77
MT	3.88	11.09	33.68	3.88	11.09	33.68	4.11	11.74	23.51
AT	1.98	5.65	39.33	1.98	5.65	39.33	3.07	8.77	32.28
VS	1.35	3.86	43.20	1.35	3.86	43.20	2.64	7.55	39.83
SB	1.26	3.59	46.78	1.26	3.59	46.78	2.43	6.95	46.78
